# A comprehensive review of mycotoxins, their toxicity, and innovative detoxification methods

**DOI:** 10.1016/j.toxrep.2025.101952

**Published:** 2025-02-07

**Authors:** Ravikant Shekhar, Vinay B. Raghavendra, P. Rachitha

**Affiliations:** Department of Biotechnology, Teresian College, Siddarthanagar, Mysore 570011, India

**Keywords:** Food mycotoxins, Detection, Toxicity, Detoxification strategies

## Abstract

A comprehensive overview of food mycotoxins, their toxicity, and contemporary detoxification techniques is given in this article. Mycotoxins, which are harmful secondary metabolites generated by a variety of fungi, including *Fusarium*, *Aspergillus*, and *Penicillium*, provide serious health concerns to humans and animals. These include hepatotoxicity, neurotoxicity, and carcinogenicity. Mycotoxins are commonly found in basic food products, as evidenced by recent studies, raising worries about public health and food safety. The article discusses detection techniques such as enzyme-linked immunosorbent assays (ELISA), and quick strip tests. Moreover, the use of various control systems associated with the detoxification of mycotoxinis highlighted. In addition, novel detoxification strategies such as nanotechnology, plant extracts, and omics studies were also discussed. When taken as a whole, this analysis helps to clarify the pressing need for efficient management and monitoring techniques to prevent mycotoxin contamination in the food chain.

## Highlights

• An overview of food mycotoxins, their toxicity, and contemporary detoxification techniques.
• Mycotoxins are commonly found in basic food products, as evidenced by recent studies.
• Updates detoxification strategies/control systems to reduce the amount of mycotoxin in food.
• In addition novel detoxification strategies such as Nanotechnology, plant extracts, and omics studies were also discussed.

## Graphical Abstracts

**Figure:**
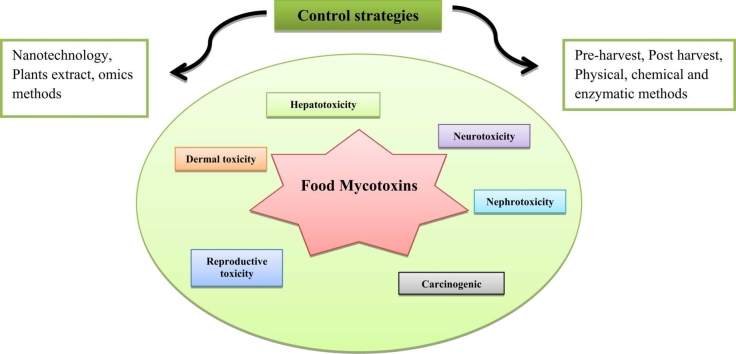

## 1 Introduction

The term ‘mycotoxin’ was first used in 1960, being derivative of two terms “myco” meaning fungus, while “toxin” means poison, hence mycotoxins are the toxic secondary metabolites that are produced by a certain class of fungi growing on various kinds of foodstuff like grains, dried fruit & nuts, and spices. The optimal growth condition for fungal growth varies between 10–40℃, pH 8.4 with a water activity (a_w_) greater than 0.70 [1]. Fungi present in the field require 70–90 % relative humidity and, a temperature of 20 – 25 ^o^C. The active growth phase of a fungi is when the fungus develops the mycelium and requires the a_w_ of 0.85 and active development requires a_w_ of 0.99. on the other hand, storage fungi are much more suited for lower humidity and higher temperatures [2]. These toxic fungi can contaminate the harvest at any point in time such as before or after the harvest or even during the storage. Long-term storage provides favorable conditions such as moisture, dietary ingredients, etc. for the growth of the fungi [3]. Being chemically stable and can resist most of the food processing processes, the presence of these toxins in the foodstuff is a major concern across the world as it harms the health of humans and animals and even contributes significantly to economic losses. A scenario in the United Kingdom where 1,00,000 turkeys died after ingestion of feed that was contaminated with the toxin produced by *Aspergillus flavus* [4].

Mycotoxicosis is a sickness caused because of mycotoxins that affect several organs and may cause death if exposed to high concentrations. The potential source for mycotoxin exposure is through the intake of contaminated food, contact, and inhalation [5]. The toxicity varies greatly with the fungi and the active chemicals present in them. This may cause endocrine abnormalities, teratogenic, mutagenic, hemorrhagic, estrogenic, hepatotoxic, nephrotoxic, immunosuppressive, altering of the gene expression, demining the reproductive system, disruption in the digestive tract and development of the cancer-causing cells in the body [6], [7].

Mycotoxins have been viewed as harmful agents to the human body even in minute levels (molecular weight, Mw 700). According to reports, mycotoxins affected 25 % of the world's grain supply, making it one of the top hazards identified by the EU's Rapid Alert System for Food and Feed (RASFF). It led to the destruction of one billion metric tonnes of food and merchandise [8].

These compounds are generally thermostable during manufacturing and food processing [9], [10], [11]. Mycotoxins can be seen under ultraviolet (UV) light; they also have no distinct odor and do not change the organoleptic properties of meals [12].

During an infection, reactive oxygen species can activate the response pathway in the fungus associated with mycotoxin production. Mycotoxins may be formed during the crop's preharvest phase and subsequently build throughout harvest, transportation, and storage [13]. These toxins might be present at any point throughout the supply chain. When adequate circumstances (moisture, temperature, and water activity) are met, the fungi can enter the food framework. As a result, it harmed human health and lowered food production profitability [14], [15], [16], [17], [18]. Foods like cereals, spices, feed, milk and dairy, nuts, and lentils are the crops most impacted by mycotoxins [19].

**Fig. 1:**
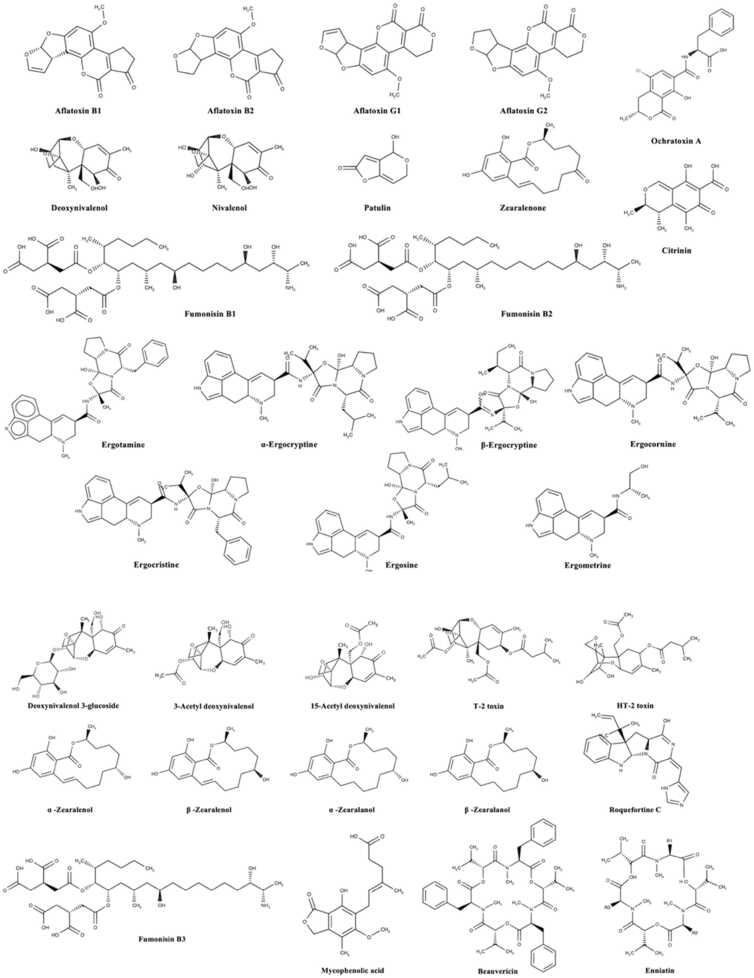
Chemical Structure of various mycotoxins affecting the food and feed [25].

A portion of the significant mycotoxins recognized incorporate Aflatoxin (AFs), present as B1, B2, G1, G2 and M1, Ochratoxins (OTs) mainly present as Ochratoxin A (OTA), Fumonisins (FBs) present as Fumonisins B1 (FB1), B2 (FB2), and B3 (FB3), trichothecenes (TCs) present in the structure with type A, represented as HT-2 toxin (HT2) and T-2 toxin (T-2) and type B represented essentially by Deoxynivalenol (DON), Zearalenone (ZEN), the arising *Fusarium* mycotoxins fusaproliferin (FP), moniliformin (MON), beauvericin (BEA), NX-2 toxin and enniatins (ENNs), Ergot alkaloids (EAs), *Alternaria* toxin (ALTs) for example, attenuate (ALT), alternariol (AOH), alternariol methyl ether (AME), altertoxin (ALTs) and tenuazonic acid (TeA), and Patulin (PAT) [13] as shown in Fig. 1.

The most vulnerable cropsto the presence of mycotoxin include maize, millet, wheat, sorghum, soybean, and peanut. The mycotoxin also affects derivatives and by-products of the infected crop. Mycotoxins are also found in food waste, moldy bread, and cotton seed, as well as cereals, leguminous seeds, and oilseeds. Industrial by-products thathave been co-ordinated with animal feed likewise contain a lot of mycotoxins and subsequently, influence the well-being of farmers, and consumers along side the animals [20]. Fungi (*Aspergillus, Fusarium, Penicillium, Alternaria, and Claviceps*), and saprophytic molds are the most well-known species that produce toxins that mischief crops all through the pre-and post-harvest period (Table 1).

**Table 1:** Various Mycotoxins and the fungal species.

**Sl. No.**	**Mycotoxin**	**Structure**	**Fungal Species**	**References**
1.	Aflatoxin		*A.bombycis*, *A. nomius*, *A. parasiticus*, *A. parvisclerotigenus*, *A. pseudocaelatus*, *A. minisclerotigenes*,	[141]
2.	Ochratoxin		*Aspergillus melleus*, *Aspergillus cabonarius*, *Aspergillus glaucus*, *Aspergillus niger*, *Penicillium viridicatum*	[138]
3.	T−2 Toxin		F. *poae,* *F. sporotrichioides*, *F.tricinctum*	[142]
4.	Deoxynivalenol		*Fusarium sp.*	[143]
5.	Fumonisins		*Fusarium proliferatum* *Fusarium verticillioide*	[144]

Several developing regions are currently at high risk for mycotoxin prevalence, ranging from severe to extreme, even though robust agricultural methods, although effective quality control and storage mechanisms are thought to lower the danger of mycotoxin exposure in industrialized countries. For instance, mycotoxins are moderate to high in South East Asia, Oceania, and Europe, but they are notably common in North and Central America, along with South Asia. Mycotoxins are surprisingly not limited to grains, concentrates, and silage, the result of free-grazing animals can likewise incorporate mycotoxins. Members of the TH and ZEA families, T-2 toxin and HT-2 toxin have been distinguished in eating grasses [21], and AFM1 has been accounted for free-grazing cow milk [22], even in past as far as possible. Therefore, it could be surmised that mycotoxin is available in all species, paying little mind to how they are raised—seriously, steadily, in the middle between. Since these frameworks rely upon rummages, yields, and animal waste, mycotoxin defilement is a risk in traveling and blended crop-animals frameworks.

Since the majority of mycotoxins are heat-stable and produce harmful breakdown products, efficient degradation of mycotoxins is difficult. Two strategies are used to manage mycotoxin contamination: prevention of their generation, and detoxification [23]. Conventional cooking methods cannot completely eradicate mycotoxins. A variety of food processing techniques together with physical, chemical, and biological procedures are employed to eradicate mycotoxins partially [24].

This review outlines primary mycotoxins and their effects on human and animals. Besides, control, anticipation, and cleaning/detoxification methodologies for food control.

## 2 Detection of mycotoxin

In the previous section, the harmful effects of mycotoxin on human health as well as on the economy were noted, hence, it is much more important to detect any kind of presence of these mycotoxins in the food and feed. Among all the chromatographic procedure is the most well-known and widely involved strategy for the recognition of the concentrates, including means like extraction, purification, and subjective and quantitative investigation.

The Association of Official Analytical Collaboration International (AOAC) has also issued several analytical procedures for the detection and analysis of mycotoxin [26]. A variety of techniques like HPLC, ELISA, Liquid-Liquid Extraction (LLE), Supercritical Fluid Extraction (SFE), Solid Phase Extraction (SPE), and Liquid Chromatography (LC) methods with UV or Fluorometric Detection (FLD), can be utilized for the illustration of the mycotoxin structure. Other chromatography-based techniques like TLC and GC are also used for the identification but the advancement in the analytical instruments has highlighted the potential of LC mass spectrometry (MS), particularly for the identification and confirmation of multiple mycotoxins [27], [28], [29], [30].

## 3 Mycotoxin control: Prevention and detoxification in food

It is vital to follow the mycotoxin control strategies to reduce the defilement in agricultural commodities. The control strategies include Pre-Harvest strategies expecting to keep away from the improvement of toxigenic growths prompting the creation of the mycotoxin; post-harvest strategies include the detoxification of the food primarily by physical or chemical control [31].

### 3.1 Pre-harvest strategies

Pre-harvest strategies include Good Agricultural Practices (GAPs), which include the restricted utilization of enlisted insecticides, fungicides, and herbicides for the control of damage caused by the respective agents. Legitimate treatment of the soil bed, and soil analysis for the assurance of the compost necessity or to search for the genetic synthesis required to suppress the mycotoxin production, also play a crucial role in the pre-harvest strategies [32], [33]. Additionally, to avoid mycotoxin contamination in common cereals, grapes, and apples, the employment of biological control agents like the utilization of the opposing growth of fungi also plays a crucial role in pre-harvest strategies. To work in concert with Hazard Analysis and Critical Control Points (HACCP) at food processing facilities, Good Manufacturing Practices (GAMs) must be followed along with the GAPs [34]. Mold development and mycotoxin production can also be reduced by controlling various environmental factors such as temperature and humidity, which are considered to have the most effects on the toxin-producing fungus. Good storage practices like regulation of the warehouse temperature, moisture content, and humidity can also significantly lessen the growth of toxigenic fungi [31].

### 3.2 Post-harvest strategies

A worldwide issue, both scientifically and practically, is the decontamination or detoxification of mycotoxins from diverse agricultural products. Elimination of the mycotoxin naturally using techniques like thermal insulation, radiation therapy, and low-temperature plasma, chemically using processes like oxidation, reduction, hydrolysis, alcoholysis, and absorption, and biologically using biological agents [35]. Detoxification including physical and chemical techniques has several drawbacks, including the loss of nutrients, the requirement for expensive equipment, and the length and effectiveness of the process. According to Wang et al. [36], biological techniques prove to be more efficient, specialized, and ecologically friendly.

#### 3.2.1 Physical treatment

Physical treatment involves several techniques to get rid of mycotoxin organically which involves drying, cleaning, segregation, washing, milling, roasting, boiling, etc. The issue of mycotoxin contamination might be exacerbated by the use of preventative post-harvest HACCP measures [13].

##### 3.2.1.1 Sorting

Unquestionably, the first stage in natural disinfection is washing and sorting. Since sorting techniques don't run the danger of generating items that degrade, they may be considered superior procedures. Following purification, the percentage of total FBs in maize reduced between 26 % and 69 % [37]. Patulin levels in fruit products may be greatly reduced to 99 % by sorting and removing decaying and subpar fruits [38]. After sorting the diseased maize, a drop in FB of 27–93 % was seen. Because an aflatoxin infection is typically heterogeneous, separating damaged nuclei from healthy ones can significantly lower infection. To minimize AFs during sorting grains, UV light was also used [39].

##### 3.2.1.2 Processing

Processing methods can lessen mycotoxins' concentrations but they cannot eradicate them [40]. Because the fungus builds up on the granules' surface, softening can lower the degree of mycotoxin contamination. In Kenya, a study showed that peeling corn reduced the number of AFs. Mycotoxins like DON and ZEN were found in significant concentrations on the granules, while the finished flour was less polluted. The quantity of mycotoxin present in the finished product might vary depending on time and temperature. Mycotoxins are much more dangerous as they are thermally stable substances, yet several common food preparation techniques like baking and frying at high temperatures may be able to reduce the mycotoxins.The extrusion process reduces the AFs by 50 %–80 % depending on the processing conditions like temperature and moisture content of the granules [41]. However, the processing in the presence of high temperatures ranging between 150 – 200 °C dramatically decreased AFB1, generating an average drop of 79 % [42].

##### 3.2.1.3 Storage

Since storage conditions have an impact on fungi's general development, they are crucial for managing mycotoxins. Conditions particularly high humidity and optimal temperature in particular might provide favorable conditionsfor fungal growth and the creation of mycotoxins. Mycotoxin’s build-up and fungal development are reduced during storage when specific parameters are met, such as proper packing, temperature management, ventilation, and air humidity [43]. Lack of proper storage led to crop losses of about 20–50 % in under developed nations [44].

##### 3.2.1.4 Radiation

Radiation in many stored grains' natural detoxifying processes. Most commonly used radiation is of two categories i.e., Ionising radiation and non-ionizing radiation [41]. Pathogenic bacteria can be reduced or completely removed by radiation; however, toxins are only partially removed from the food. Radiation transfers the energy leading to slight modification in the molecular structure of the foodstuff through a series of reactions and can be utilized on the industrial level [39]. Accessible literature suggests that ZEA toxins were diminished fundamentally and the radiation was protected up to an illumination of 10 kGy in irradiated refined water and natural products like orange, pineapple, and tomato juices. The fruit juices' quality was impacted by a greater radiation dosage [45]. In recent research by Zhong et al. [38], ZEN and OTA levels in contaminated corn decreased by about 71.1 % and 67.9 % as a result of electron beam irradiation of 50 kGy. Additionally, upon gamma irradiation when employed to treat rice, AFB1 was reduced by more than 95 % (at 6 kGy) [43]. PAT was significantly reduced to about 83 % after 5 minutes of irradiation in apple juice [46]. Radiation was suggested as a possible method for mycotoxin detoxification, but due to the possibility of physical, chemical, and biological side effects as a result of probable molecular interactions, its usefulness is still in question [47].

##### 3.2.1.5 Cold Plasma (CP)

It is utilized to kill bacteria since it has potent antimicrobial properties. The term plasma is used to refer to the fourth form of matter, which contains mostly ions, and free radicals with distinct physical and chemical characteristics, such as reactive oxygen and nitrogen species. Cold Atmospheric Pressure Plasma (CAPP) techniques can be used as an alternative method for the defiling of mycotoxin, which is promising, affordable, and ecologically benign. However, the surface of nuts was detoxified by 50 % using low-pressure cold plasma [48]. Since no investigation on the potential creation of hazardous chemicals was done, this approach needs to be used with caution. After only 10 minutes of treatment, CAPP significantly reduced AFB1 and FB1 mycotoxins by up to 66 % in maize. Additionally, after 8 minutes of exposure, cold atmospheric plasma use resulted in a reduction of Afs, TCs, ZEA, and FUs of 93 %, 90 %, 100 %, and 93 % respectively. AFB1, DON, and NIV degraded completely after only a 5-second plasma treatment [39], [41], [49], [50].

##### 3.2.1.6 Mycotoxin Binders

This prevent mycotoxins from being absorbed because they bind to them and prevent them from entering the bloodstream through the stomach. Activated carbon, aluminosilicates, complicated non-digestible polysaccharides, and cholesterol are some examples of absorbent materials [51]. An alternate physical method to the microbial destruction of AFs is by using the binding to the mycotoxin. Microbiological enzymes may target the lactone ring for cleavage, which lessens the toxicity of AFs [52]. Mycotoxin levels were decreased, however more research is required to guarantee food safety [39].

#### 3.2.2 Chemical Control

##### 3.2.2.1 Bases (Ammonia, Hydrated Oxide)

A variety of mycotoxins (AFs, FBs, and OTs) are reduced to undetectable levels when seeds are treated with ammonia, and the development of mycotoxigenic fungus is suppressed. However, in the EU, base treatment of food meant for human consumption is prohibited. Mycotoxin detoxification was greatly aided by using glycerol and calcium hydroxide solution [53]. Although these compounds have the potential to lead to secondary contamination and have negative effects on the nutritional content of the goods, they are frequently utilised in the breakdown of AFB1 in contaminated oil [54].

##### 3.2.2.2 Chitosan

After cellulose, chitosan is a linear polysaccharide that is abundant in nature and inhibits fungus, bacteria, and viruses. Chitosan is particularly intriguing for the food preservation due to its biocompatibility and antibacterial capabilities [55], [56]. The consolidate impacts of chitosan and a_w_ for controlling the contagious development and mycotoxin production of FBs and DON by the Fusarium species (*F. proliferatum*, *F. graminearum*, and *F. verticillioides*) were reported on maize and wheat, showing a decline in DON and FB production in irradiated maize and wheat grains following the utilization of low-sub-atomic-weight chitosan with deacetylation above 70 %, and a portion of 0.5 mg/g [56]. Furthermore, the treatment of 1 % chitosan improved with 1 % lemon medicinal oils in figs diminished the degrees of DON in wheat grains from seaside brown algae *Ascophyllum nodosum* [57].

##### 3.2.2.3 Ozone treatment

Ozonation is a simple process that, when used, leaves no toxic residues behind. Ozone is used for detoxification of mycotoxins or to sterilise grains, vegetables, and fruits [58]. Quintanilla-Villanueva et al. [59], claimed that since AFB1 and AFG1's structures include a C8-C9 double bond, ozone gas is particularly effective at degrading aflatoxins. AFG1 in particular turned shown as the most susceptible. Under ideal circumstances, ozone treatment resulted in large reductions in DON (29 %–32 %) and DON-3-glucoside (DON-3-Glc) (44 %), which is the modification of DON. Additionally, a notable microbial reduction was seen in durum wheat, which had no effect on the chemical and rheological characteristics of the semolina and pasta prepared out of ozonated wheat [60]. DON was changed into 10 ozonized products (C_15_H_18_O_7_, C_15_H_18_O_9_, C_15_H_22_O_9_, C_15_H_20_O_10_, C_15_H_18_O_8_, C_15_H_20_O_9_, C_14_H_18_O_7_, C_14_H_16_O_6_, C_15_H_20_O_7_, and C_15_H_20_O_10_) after treatment with ozone in the gaseous form [61].

The rate of DON degradation was positively associated with both the treatment time and ozone concentration, for example, 30 s treatment with an ozone concentration of 1 mg/L, the rate of destruction of DON in solution was achieved to be 54.2 %. The granules' moisture concentration has a considerable impact on DON decomposition. When ozone concentrations of 60 mg L^-1^ were administered for 12 hours to wheat having 17.0 % of moisture content, the rate of DON breakdown was 57.3 % [58]. Fresh noodles created from ozone-treated wheat flour preserved more in terms of microbial development, according to research by Wang et al., [62].

#### 3.2.3 Biological Control

Numerous studies from teams with various backgrounds and levels of research expertise have achieved significant advancements in the previous 20 years in the quest to find a suitable biological agent for the detoxification of mycotoxin [63]. Numerous studies have documented the usage of microorganisms such bacteria, yeast, and fungus for the detoxification of mycotoxin in food and feed [64], [65], [66]. A different strategy for controlling mycotoxins is to detoxify/degrade them biologically, which can result in the generation of limited harmful end products. The in vitro decontamination of mycotoxins was significantly aided by pure strains of microbes. Additionally, fermentation's efficiency in lowering and getting rid of mycotoxins has been proven [67].

##### 3.2.3.1 Bacteria

Mycotoxins in meals or drinks can be bound by certain bacteria [66] *Flavobacterium aurantiacum* B-184 was the only bacteria out of more than 1000 examined for potential AF degradation that could permanently remove aflatoxin from solutions. By attaching to the components of the bacterium's cell wall, AFB1 is detoxified by *Enterococcus faecium*. Mycotoxins have been found to attach to bacterial cell walls' peptidoglycans and polysaccharides with the assistance of microbes [68]. Furthermore, because of research efforts and advancements, microorganisms gained the ability to detoxify the mycotoxin DON. Solutions to lessen DON pollution are provided by the aerobic based oxidation and partitioning of DON into C3 carbon transported by several species of *Devosia* [63]. In aqueous solutions, the lactic acid bacteria like *Lactobacillus casei* and *Lactobacillus reuteri* were successful in binding to AFs. Other in vitro assays revealed a binding effectiveness of up to 60 % AFB1, demonstrating the ability of *Lactobacillus amylovorus* and *Lactobacillus rhamnosus*to bind certain dietary pollutants [69]. Additionally, during the fermentation of whole-grain sorghum with Lactobacillus fermentum, decreases of 98 % FB1 and 84 % T-2 was achieved [70].

##### 3.2.3.2 Yeast

The employment of competing yeasts is particularly interesting since yeasts can grow quickly on a number of substrates in bioreactors and create antimicrobial chemicals that have positive effects on people and animals. Additionally, yeasts do not create allergens or other secondary metabolites, in contrast to many filamentous fungus or bacterial adversaries [71], [72]. A probiotic yeast called *Saccharomyces cerevisiae* significantly break down DON and slow the production of lactate dehydrogenase (LDH) in DON-stimulated cells [73]. Additionally, the inclusion of *S. cerevisiae* yeast cell walls can lower low amounts of the mycotoxins AFB1 and OTA in chicken diets [74]. Additionally, the efficiency of lowering the mycotoxin patulin in fermented foods by *S. cerevisiae* by lengthening the fermentation period and raising the temperature was examined. Physical adsorption is a method that yeast cells can use to get rid of PAT. In actuality, PAT interacts with the O-N/N-H protein and polysaccharide linkages of cell walls [75]. Mycotoxins AFB1, OTA, or ZEA were bound by *Kluyveromyces marxianus*. The findings demonstrated that mycotoxins, particularly those that affect *C. utilis*, may attach to cell membranes [76]. In a different investigation, the yeast *Yarrowia lipolytica* reduced the level of OTA to almost half of what was first added to the colony [77]. Additionally, PAT was broken down by yeast strain *Rhodotorula mucilaginosa* (JM19), and was analysed using HPLC-UV. Dexipitulic acid was the product from the breakdown of PAT. PAT debasement by *R. mucilaginosa* JM19 was fundamentally affected by the temperature, cell density and seeding concentration of PAT. After 21 h at 35 °C and when the density of yeast cells was more than 1 × 10^8^ cells/L, a 90 % decrease in PAT was noticed. At an underlying PAT concentration of 100 μg/mL, *R. mucilaginosa* JM19 was found to be well suited for causing more than 50 % degradation, referring to its value in debasement of PAT in foods and raw materials [78].

##### 3.2.3.3 Food Fermentation

Foods undergo fermentation, which raises their quality while giving them qualities that are very appealing to customers. Fermentation is a very cheap method of mycotoxin disinfection that may be employed for the enhancement of food components and also to lessen or even get rid of mycotoxins. As an alternative to expensive and impracticable methods, fermentation can be a desired method to minimise mycotoxins. In terms of production of safe foods, the type of metabolites and the toxicity of products created following fermentation should be well characterised [67].

##### 3.2.3.4 Fungi

On maize, cotton, pistachio, and peanuts, the reception of anon-toxic strains of *A. flavus and A. parasiticus* brought about outstanding viability in lessening aflatoxin pollution. As per the available literature on organisms and their detoxification, growths that might create aflatoxin can likewise separate them. This is due to the fact that these fungi may often degrade, perhaps convert, and utilise degradation products as a source of energy during starvation [79]. Fungi such as *Aspergillus*, *Rhizopus, Trichoderma, Clonostachys,* and *Penicillium* spp. show effective capacities for the detoxification of mycotoxins. The biocontrol of AFs in maize with non-toxic microbial strains depends up on competition in both west and east Africa. Especially immense amount of non-toxic *A. flavus* and *A. parasiticus* inoculants contend with toxigenic strains in the soil around the crops [67].

### 3.3 Enzymatic detoxification

Mycotoxins are processed chemically and biologically during enzymatic detoxification. It can be used in multiple settings, has a high level of performance and specialisation, is non-toxic to living things, and is applied under benign circumstances. Furthermore, non-stoichiometric ratios of mycotoxins include enzymes acting as catalysts [80]. Some *Aspergillus* species have produced an enzyme that has the inherent ability to detoxify fumonisins, which are produced by *Fusarium* [81]. Depending on the properties of the microorganism, some enzymes, such as chitinase and β 1,3-glucanase, may have different anti-pathogen activities. Use of -β 1,3-glucanases and chitinases has an impact on the delay and reduction in development of fruit rotting fungus. Spraying β 1,3-glucanase at a concentration of 50 % and chitinase at the concentration of 50 % and 40 % caused *Penicillium simplicissimum*, *A. niger* complex, *Penicillium nalgiovense,* and *A. flavus* growth was ceased over the surface of salami. In order to prevent fungal spoiling in the fermented sausage sector, β 1,3-glucanase and chitinase proved to be an alternatives [82]. AFB1 was also subjected to enzymatic detoxification using microbial manganese peroxide, oxidase enzymes, catalase, and laccase enzymes. However, due to their advantageous toxicity and specialisation, enzymes have an unknown profile for detoxifying dietary toxins. No enzyme is permitted in the EU to remove mycotoxin contamination from food [41], [67].

### 3.4 Novel detoxification strategies

#### 3.4.1 Nanoparticles

Numerous researches suggested removing mycotoxins with the helpful adsorbents of nanoparticles. Many researchers have reported that chitosan-coated Fe_3_O_4_ nanoparticles were utilised for PAT decontamination, silver nanoparticles was employed for the destruction of *Fusarium* spp. and their principal related mycotoxins, and magnetic carbon nanocomposites were employed for AFB1 detoxification [38], [83]. UCNP@TiO_2_ (up-conversion nanoparticle), a novel phyto-catalyst was created and employed to break down DON, claims the recent research. The findings revealed that DON levels in cereal goods dropped below the allowable limits (1 ppm) after 90 minutes and completely degraded after 120 minutes of illumination. The breakdown products of the UCNP@TiO_2_ composite material were barely hazardous or even non-toxic, making it effective and environmentally friendly. As a result, mycotoxin detoxification may be accomplished using this degrading method [84]. González-Jartn et al. [85] observed that nanocomposites made of combinations of activated carbon, bentonite, and aluminium oxide eliminated up to 87 % of mycotoxins.

##### 3.4.1.1 Plant Extracts

Numerous essential oils (EOs) and the primary bioactive components in them have been employed for their antifungal and anti-mycotoxigenic activities [86], [87], [88], and it has been shown that they can stop certain mycotoxins from being produced [89]. Because they are thought to be safe for humans and beneficial to the environment, botanicals are typically preferred to chemical treatments in the elimination of toxic fungus and mycotoxins. According to several studies, the essential oils of turmeric and eugenol, the main component of clove oil, as well as *Aspergillus* growth and AFB1 synthesis are all inhibited. The utilization of entire cloves in culture media and rice grains diminished the growth of *A. flavus* and *P. citrinum* as well as the production of their toxins [38], [90].

In a recent investigation, it was reported that the Spanish paprika smoker "Pimentón de la Vera" had an impacted the growth of *A. parasiticus* and *P. nordicum* as well as the generation of AFB1, AFG1, and OTA. The growth and generation of the mycotoxins AF and OTA were declined by the application of 2 %–3 % Spanish paprika smoker to meat products as fillets or sausage preparations [91]. Additionally, the natural ingredient capsaicin reduced the amount of OTA produced in grapes by *A. carbonarius* by 61.5 % and *Aspergillus niger* strains by 28.9–78.1 % [92].

### 3.5 Omics studies

#### 3.5.1 Genomics approach

Genomic approach for the study of the mycotoxin is a urgent cycle in grasping the design, capabilities, and communications of an organic entity's hereditary material. It includes describing the design of an organic entity's genome, contrasting it and related life forms, and recognizing the capabilities and co-operations of incorporated proteins or qualities [94]. The main complete DNA arrangement was acquired in 1992 from *Saccharomyces cerevisiae*, and the human genome was finished in 2004 [95]. Useful genomics assists scientists with understanding the communication among organisms and host plants, giving knowledge into plant-contagious quality connection and mycotoxin creation [96].

The genomics approach can be viewed as a pre-collect application to recognize qualities liable for mycotoxin creation. A few genomic studies have been directed on mycotoxigenic growths, especially on aflatoxin-delivering *A. flavus* [93]. The first genomic investigation recognized more than 7200 interesting EST groupings, while the J. Craig Venter Organization finished the investigation utilizing progressed bioinformatics methods [97].

Different genomic instruments, for example, Particle Downpour Individual Genome Machine (PGM), microarray examination, and quantitative converse record PCR (qRT-PCR), have been utilized to distinguish qualities liable for mycotoxin creation and foster screening strategies. The genomic approach likewise considers the ID and separation of mycotoxin-creating qualities in wild and freak strains [98], [99].

*Aspergillus flavus* is a critical animal groups in the comprehension of aflatoxigenic qualities, biosynthetic pathways, and aflatoxin digestion. It has been viewed as exceptionally like other *Aspergillus* species, for example, *A. oryzae*, which has a critical monetary effect because of its utilization in food maturation processes. DNA sequencing of field contagious detaches and correlation with the quality arrangement of aflatoxin-delivering *A. flavus* is significant for distinguishing objective qualities and controlling aflatoxin creation in crops [99], [100], [101].

In 2014, 240 *Aspergillus* strains from nut seeds were confined and classified into nine clade. A review utilizing practical genomics surveyed the effect of environmental change on *A. flavus* and aflatoxin creation, uncovering that worldwide temperature, water accessibility, and rising CO_2_ levels influence the outflow of the aflatoxin biosynthetic administrative quality aflR [102], [103], [104].

Late examinations have zeroed in on the quality grouping of ochratoxin A (OTA) delivering species to recognize qualities liable for creating OTA. Ochratoxin A biosynthesis was before hand obscure, however another understanding was given through erasure of a non-ribosomal peptide synthetase quality in *A. carbonarius*. A new complete genome grouping of filamentous parasite *A. westerdijkiae* outlined the potential biosynthetic quality bunch of OTA and the genome of *A. westerdijkiae* [101], [105], [106].

Patulin biosynthesis is still being scrutinized with omics apparatuses, and ongoing investigations have recognized two types of *Penicillium* spp. creating patulin. Be that as it may, the total genome for *A. clavatus* is as yet not accessible [107], [108].

#### 3.5.2 Transcriptomics approach

Transcriptomics is an as of late evolved field that spotlights on grasping quality articulation and cell capabilities. It includes concentrating on the total arrangement of RNA records delivered by qualities under unambiguous circumstances from a particular tissue or cell type. Transcriptome examination permits specialists to comprehend the statement of the genome at the record level, giving data on quality construction, protein alterations, elements of integrated quality items, and developmental changes of end organic cycles [109], [110], [111], [112].

Qualities direct and communicate in various organic and physiological circumstances, prompting various proteins being blended. Transcriptomics and high level scientific devices assume a significant part in grasping complex natural frameworks and creating novel biomarkers. It has the potential for beginning phase analysis and viable medicines in the restorative or rural industry [113], [114].

Transcriptional profiling is the far-reaching investigation of the total arrangement of RNA records from the genomes of a cell, tissue, or living being. Strategies, for example, Northern smudges, nylon film clusters, switch transcriptase quantitative PCR (RT-qPCR), and sequential examination of quality articulation (SAGE) are utilized to concentrate on RNA records [115], [116], [117].

Transcriptomic concentrates on aflatoxins, ochratoxins, and patulin explore the organism plant crosstalk, abiotic factors influencing mycotoxin creation, and poisonousness instruments. Studies have recognized qualities of interest engaged with the cross-species organization, like those associated with aflatoxin creation and vascular vehicle [118], [119].

All in all, transcriptomics and high-level scientific instruments assume a huge part in figuring out complex natural frameworks and creating novel biomarkers for early determination and treatment in the rural and restorative businesses.

#### 3.5.3 Metagenomics approach

The high-throughput sequencing procedures give an incredible asset to evaluate the effect of the microenvironment in crops on the "Mycobiome". Ongoing distributions have featured the convenience of those instruments [120], [121], [122]. Analyzed peanuts put away for 90 days at 20–30 °C and relative humidities of 70, 75 and 80 %. Sequencing investigation uncovered a general abatement of contagious functional ordered units (FOU) all through stockpiling and an expansion in *A. flavus* relative overflow all through the capacity period, autonomous of the circumstances tried [123].

**Fig. 2:**
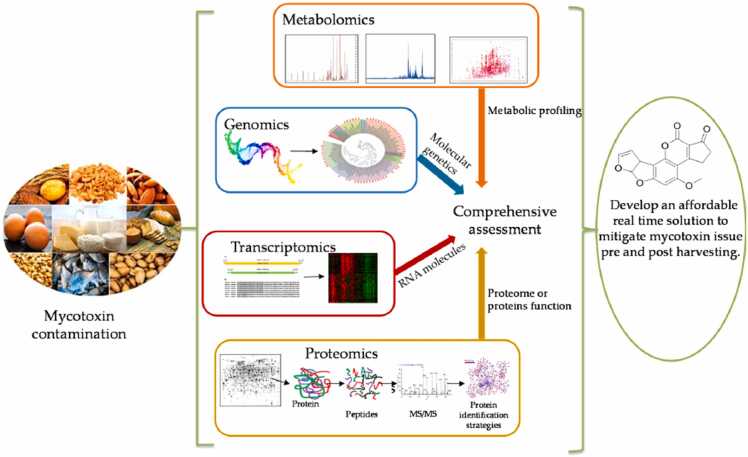
Overview of the “omics” approach in the mycotoxin studies [93].

Future exploration on fleeting metagenomics and transcriptomic information in 3-layered natural collaborating pressure factors (temperature, pH and CO_2_) ought to give advancement information to translate the parasitic collaboration in crops and the strength of different parasitic networks. This would be helpful in figuring out these progressions and furthermore distinguishing advantageous microorganisms, for instance potential biocontrol up-and-comers in unambiguous biological specialties for mycotoxin control. These methodologies will require equal improvement of hearty bioinformatics apparatuses to accumulate information and characterize designs because of interfacing natural pressure factors (Fig. 2).

#### 3.5.4 Proteomics Approach

In the investigation of plant pathogenic parasites, the principal concentrates on utilizing a proteomic approach were completed in 1980s with the point of a superior comprehension of plant-contagious microorganism connections through the hunt of opposition related proteins [124]. Not with standing, albeit a few examination studies have involved proteomics to concentrate on the science what's more, pathogenesis of parasitic plant microbes (e.g., *Blumeria graminis f. sp*. *hordei*, *Botrytis cinerea, Leptosphaeria maculans, Magnaporthe grisea, Ustilagomaydis, Phytophthora infestans*), including mycotoxigenic growths (*A. flavus; F. graminearum*), the impact of collaborating natural changes on transformation and poison creation hasnot been generally considered [125], [126].

Ramirez-Garcia et al. [127] looked at the secretome of *A. niger* when xylose or maltose were enhanced in the medium. That's what their outcomes showed albeit the secretome was firmly influencing the intracellular proteome, this was not fundamentally different. In contrasts in culture conditions (pH control versus no pH control, air circulation versus no air circulation and blending as opposed to shaking) significantly affected the intracellular proteome.

Wang et al. [128] portrayed the impact of the consolidated expansion of lactate and starch in the mode for the development of *A. niger*. The outcomes showed that fumonisin B2 creation was essentially expanded. The proteome of *A. niger* was plainly unique during development on media containing 3 % starch, 3 % starch + 3 % lactate or 3 % lactate. A considerable lot of these were chemicals engaged with essential digestion and different cycles that influence the intracellular degree of acetyl-CoA ornicotinamide adenine dinucleotide phosphate (NADPH).

The impact of various light frequencies on differentially communicated proteins delivered by *Penicillium verrucosum* filled either in obscurity or under light with a frequency of 450 nm was broke down [129]. They recognized 46 fundamentally differential proteins (light versus dull) involving proteins of a wide scope of isoelectric focuses and atomic masses. Most proteins were engaged with reaction to push (for example antioxidative proteins, heat shock proteins) and general metabolic cycles (for example glycolysis, ATP supply). Besides, the outcomes demonstrated that light of short frequency prompted oxidative pressure in the contagious cell and under this condition the mycotoxin biosynthesis uncovered a shift from ochratoxin A to citrinin.

The impact of aw on the proteomic profile of *A. flavus* was explored by Jia et al. [130]. A sum of 3566 proteins were distinguished, of which 837 were differentially communicated in light of varieties in a_w_. Among these, 403 were over-communicated at 0.99 a_w_, though 434 were over-communicated at 0.93 a_w_. Two proteins (AFL2G_04330 what's more, KapK) were recognized as playing a basic part in the enlistment of aflatoxin biosynthesis. Li et al. [131] researched the progressions in record and relative protein levels in light of temperature, corresponding transcriptomic and proteomic examinations were utilized to recognize changes in *A. flavus* developed at 28 °C and 37 °C. A sum of 3886 proteins were distinguished, and 2832 were dependably measured. Curiously the creators brought up that there was a low relationship between's the proteome and transcriptome information, proposing that post-transcriptional quality guideline may play a vital part on various organic pathways and optional metabolite quality groups.

#### 3.5.5 Metabolomics approach

Metabolomics have been depicted as exceptionally valuable in giving solid information about mycotoxin utilization in a host. It is also accounted for to give a decent degree of information on likely communications with other biomolecules and designated organs, permitting for the displaying of intraspecies changeability and biochemical crosstalk [132]. This high-throughput method followed in the metabolomics approach can give an extensive outline of the pathophysiological state intervened by mycotoxin openness, in this way assisting with finding and characterize the most applicable openness biomarkers connected with antagonistic ramifications [133]. The omics advancements in light of High Resolution Mass Spectrometry (HRMS) permit specialists to consider every one of the three likely types of existing mycotoxins, specifically, unmodified (parent) types of mycotoxins biosynthesized by various parasites (e.g., AFs, OTA, Harmony, FBs, PAT, and DON), followed by lattice bound mycotoxins, for example, those non-covalently complexed with proteins and polysaccharides, and synthetically or naturally adjusted mycotoxins (veiled or on the other hand stowed away) delivered by fungi, microbes, plants, or creatures or got from various food handling conditions [134].

Metabolomics is currently viewed as an extremely strong innovation to guarantee the unprejudiced and by large assurance of a few metabolites (i.e., small metabolites with a mass reach from 50 to 1500 Da). Consequently, this approach is tracking down wide application in various food networks [132]. The most far and wide insightful devices, it is feasible to list Nuclear Magnetic Resonance (NMR), trailed by GC and LC-HRMS. High-resolution mass analyzers take into account a very high putative recognizable proof level, for instance, utilizing Quadrupole time-of-flight (QTOF) and Fourier change based instruments (e.g., Orbitrap and particle cyclotron resonance) [132], [133], [134], [135].

Metabolomics has also uncovered a vital job of oxidized glutathione in milk tests related with the silage bunch tainted by arising *Aspergillus* toxins. Recently, the mixture of stable isotopes with metabolomics permitted for the identification of recently recognized biotransformation items in human cell models, for example, ZEN pyridoxine, DON 3-sulfate, DON 10-sulfonate 2, DON 10-glutathione, and DON cysteine, in this manner working on the best in class and existing data about the biotransformation of ZEN and DON [136], [137].

Various other examples, including rumen liquid, blood, and milk, gathered from dairy cows were recently examined by Wang et al. [138] to get an extensive comprehension of the metabolic changes coming about because of AFB1 openness. Especially, the creators joined ^1^H NMR spectroscopy with traditional biochemical tests and illustrated a huge effect of AFB1 openness on amino corrosive digestion (basically phenylalanine) in every one of the three biofluids. Nonetheless, the creators inferred that mycotoxicity ought to be better surveyed by utilizing biomarkers of metabolomics as well as certain signs of milk sythesis and creation factors. One more intriguing review was created by Ogunade et al. [139] assessing biomarkers of aflatoxin ingestion through a ^1^H NMR-put together metabolomics approach with respect to dairy cows took care of aflatoxin B1 no matter what sequestering specialists. The creators found that AFB1 extraordinarily impacted the plasma metabolomics of lactating Holstein dairy cows. Specifically, a solid reduction in plasma amino acids (like alanine, leucine, and arginine) and acidic corrosive was noticed, while a comparing expansion in ethanol was distinguished. The last option was proposed as a decent competitor biomarker of aflatoxin ingestion in dairy cows [139]. One more important illustration of a metabolomics-based approach managing mycotoxins and biofluids is addressed by a review completed by Gerdemann et al. [140] for the examination of cell modifications brought about by 20 mycotoxins in HepG2 cells. Hydrophilic-based chromatography joined with designated HRMS took into consideration the particular and delicate recognition of in excess of 100 metabolites, handily connected with metabolic changes brought about by mycotoxins. Specifically, the creators detailed moniliformin and citrinin as the most fundamentally impacted compounds for the citrus extract cycle, likewise affecting glycolysis and energy digestion. Penitrem A, Harmony, and T2 poisons predominantly resolved a lopsidedness of the urea cycle and amino corrosive homeostasis. The arrangement of ROS was related with the presence of T2 poisons and gliotoxin, while OTA modified glycolysis. At long last, DNA blend was impacted by a few mycotoxins.

### 3.6 Effectiveness and limitations of detoxification strategies

The detoxification strategies of the mycotoxin from food and feed are broadly divided into traditional approach and noval approaches. The traditional approaches include the detoxification by the utilization of the biological (using microorganisms and enzymes), and chemical (bases, ozone, chitosan etc); while the noval approach include the detoxification using nanoparticals, plant extracts, pulse electric field (PEF), uning omics studies, etc. [7].

The factors such as easy to use, cost effectiveness of the process and the addition of the nutrative value makes the traditional detoxification strategies a preferential choice of utilization to reduce the mycotoxin from the food stuff [13]. Even though the traditional method are widely used it is partially effective in the control of mycotoxin because of physical constrains such as the understanding of the relationship between the microorganisms and enzymes produced by them for the detoxification is very limited as well as at the commercial level the implementation is vey complex and lack the practicality [145].

The noval approach for the detoxification of the mycotoxin is very effective against majority of the mycotoxins and the fungal species producing them, but he complexcity in the production/synthesis process as well as utilization of the expensive tools and techniques limits their utilization by the farmers and small scall food processing industries [146].

## 4 Aflatoxin

*Aspergillus flavus* and *Aspergillus parasiticus*, are the two toxigenic forms of mould that thrive in soil, hay, decaying plants, and cereals, create aflatoxins as by-products. Acute or long-term exposure to aflatoxin can cause poisoning. “Aflatoxin” has been originated from the *A. flavus* . One of the main categories of mycotoxins is the aflatoxins. Fungal activity occurs throughout the production process i.e., from farm to feed, even during the storage, which results in the synthesis of aflatoxin. It is regarded as an inevitable food contamination by the US Food and Drug Administration (FDA). Aflatoxin exposure can result in immediate symptoms including nausea, vomiting, cramping, and convulsions, as well as chronic problems like hepatotoxicity, immunotoxicity, and teratogenicity. One of the main causes of hepatocellular cancer in poor nations is aflatoxin [147].

It has a significant negative impact on both human and animal health and is to blame for the loss of billions of dollars to the global economy by polluting various crops including cotton, peanuts, maize, and chilies. Aflatoxin types B1, B2, G1, and G2 are the most common and fatal of the more than eighteen distinct kinds that have been identified so far [148].

### 4.1 Source

*A. bombycis*, *A. nomius*, *A. parasiticus*, *A. parvisclerotigenus*, *A. pseudocaelatus*, *A. minisclerotigenes*, and *A. arachidicola* produce all four types of aflatoxins, viz. B1, B2, G1, and G2; whereas, *A. flavus*, *A. ochraceoroseus*, and *A. rambellii* only produce Aflatoxin B1 and B2; and *A*. *pseudonomius*, *A. pseudotamarii*, *Emericella astellata*, *E. olivicola*, and *E. venezuelensis* only produce aflatoxin B1 [141].

### 4.2 Occurrence

In crop such as cocoa, spices, figs, rice, wheat, maize, sesame seeds, millet, and groundnuts, aflatoxin-related contamination by fungus can happen during processing and even at pre and post harvesting process [90].

### 4.3 Toxicokinetics

Aflatoxin enters into the humans via ingestion and get converted into reactive epoxide by the action of mixed function oxidase (MFO), a CYP450 superfamily enzyme present in the liver. This epoxide intermediate (8,9-epoxide) causes DNA mutation by the GT transversion at codon 249 of p53, a Tumour suppressor gene.This epoxide can cause cellular dysregulation via getting attached RNA and proteins. Additionally, it prevents the translation of proteins. Depletion of glutathione and reactive oxygen species are two further pathways to toxicity. Studies are being done on additional carcinogenesis pathways that are catalysed by lipid peroxidase (LPO) and prostaglandin H (PGH) synthase [149].

The hydroxylation of AFB1 by microsomal biotransformation is another method of aflatoxin metabolism. AFM1 and AFQ1, are two less harmful and non-polar metabolites, are produced as a result. AFB1 may also take on enzymatic and non-enzymatic activity that results in the di-aldehyde form. Aflatoxin aldehyde reductase (AFAR) converts aflatoxin di-aldehyde to di alcohol, which is then eliminated through the urine. It may bind proteins as well, primarily albumin [150].

### 4.4 Detoxification strategies

#### 4.4.1 Probiotics

Aflatoxin contamination of food items is acknowledged as a significant risk to food safety globally because to its negative impact on human wellbeing, including cancer, mutagenesis, and immunosuppression. Aflatoxin B1 (AFB1), one of several forms of aflatoxins, is very much prevalent and dangerous foodborne mycotoxin for people. To minimise the toxic effects of AFB1, a variety of detoxifying techniques are employed. The efficacy of probiotics isolated from "laban" (a fermented milk beverage), "idli" batter (fermented rice and black gramme), and yoghurt (made by bacterial fermentation of milk) to detoxify AFB1 was examined in the current investigation.The isolate YGT1 from yoghurt demonstrated the greatest (83.8 %) degradation of AFB1 in Luria-Bertani (LB) liquid medium after 48 hours of incubation at 30 °C among the four isolates from fermented foods examined. A further confirmation of the probiotic isolate's breakdown of AFB1 came from a liquid chromatography/mass spectrometry assay. *Bacillus subtilis* YGT1, bacterial isolate based on examination of the 16S rRNA gene sequence. The breakdown of AFB1 was also seen in the culture supernatant and heat-treated culture supernatant (boiled for 30 min) of *B. subtilis* YGT1. This indicates that thermostable bioactive compound(s) may have been involved in the degradation of AFB1. These findings showed that the yoghurt-isolated *B. subtilis* YGT1 may be a suitable candidate for use in the food and feed sectors for the elimination of AFB1 [151].

#### 4.4.2 Aflatoxin detoxification by plant extracts/active compounds

A burgeoning field of research aims to improve food safety and lower health hazards associated with mycotoxin contamination by studying the detoxification of aflatoxins, namely aflatoxin B1 (AFB1). Direct binding, metabolic transformation, and fungal growth suppression are some of the processes involved in the detoxification process. Aflatoxin-producing fungus like *A*. *flavus* and *A*. *parasiticus* can be inhibited in their growth by the antimicrobial qualities of plant extracts [152]. Effective phytochemicals found in certain plant species have shown promise in the detoxification of aflatoxin. Ajowan, garlic, ginger, and *Moringa oleifera* are a few plant extracts that have shown promise in the detoxification of aflatoxin [153], [154], [155], [156].

Applications of plant extracts for aflatoxin detoxification in the real world are found in a number of industries, such as animal feed, food processing, and agriculture. By adding certain plant extracts to food items as a natural preservative and lowering the mycotoxin concentration, food preservation can be accomplished [157]. Adding detoxifying plant extracts to animal feed reduces the possibility of mycotoxin transmission to food items made from these animals and shields livestock against aflatoxin exposure.

Aflatoxin can be rendered inactive by phytochemicals present in plant extracts. For instance, polyphenolic chemicals present in medicinal plants have the ability to chemically alter aflatoxins' structures, reducing their toxicity or aiding in their removal [158]. By improving metabolic pathways involving phase II detoxifying enzymes like glutathione-S-transferase, biochemical transformation helps turn aflatoxins into less toxic metabolites [159]. According to studies, plant extracts can boost these enzymes' activity and encourage the excretion of aflatoxins. Using plant extracts as preharvest treatments to lessen fungal infestations on crops is another way to enhance agricultural methods [160]. AFB1 contamination levels in harvested grains are decreased when crops are treated with garlic or ginger extracts, according to case study evidence [161].

#### 4.4.3 Aflatoxin detoxification using nanoparticles

Aflatoxin detoxification using nanotechnology provides novel approaches via a variety of methods. Activated carbon and graphene oxide nanoparticles efficiently absorb aflatoxins because of their porous nature and large surface area [162], [163]. The removal of aflatoxins from contaminated substrates is one way that these nanoparticles improve food safety. They have potent antifungal qualities as well, preventing the development of fungi that produce aflatoxin [164]. Magnetic nanoparticles make it simple to remove mycotoxins from food items by applying an external magnetic field, which improves detoxification effectiveness and lowers the possibility of contamination [165]. Aflatoxin contamination and fungal development are also avoided by using nanotechnology into food packaging materials. Aflatoxin contamination in grains is considerably decreased by packing loaded with silver nanoparticles, according to studies. Aflatoxin detoxification using nanotechnology provides novel approaches via a variety of methods [166], [167]. Activated carbon and graphene oxide nanoparticles efficiently absorb aflatoxins because of their porous nature and large surface area. The removal of aflatoxins from contaminated substrates is one way that these nanoparticles improve food safety [164]. They have potent antifungal qualities as well, preventing the development of fungi that produce aflatoxin. Magnetic nanoparticles make it simple to remove mycotoxins from food items by applying an external magnetic field, which improves detoxification effectiveness and lowers the possibility of contamination. Aflatoxin contamination and fungal development are also avoided by using nanotechnology into food packaging materials [168]. Aflatoxin contamination in grains is considerably decreased by packing loaded with silver nanoparticles, according to studies [169].

#### 4.4.4 Proteinase K, SDS & TCA Treatment

The most prevalent mycotoxin with the highest toxicity, aflatoxin B1 (AFB1), poses a risk for contaminating food. *P. aeruginosa* M-4, a research subject, was shown to degrade AFB1 at a rate of 56.79 %. Proteinase K, sodium dodecyl sulphate, and trichloroacetic acid treatments all significantly reduced the AFB1 degradation rate in the culture supernatant to corresponding values of 20.71 %, 10.83 %, and 19.62 %. Additionally, heating the culture supernatant for 30 minutes at 121 °C caused the rate of AFB1 degradation to increase to 84.76 %, demonstrating that extracellular proteins or enzymes were effectively degrading AFB1. *P. aeruginosa* M-4 decreased (90.57 %, 14 days) the amount of AFB1 in infected maize. The biotransformation of AFB1 into the structurally distinct molecules (C_17_H_16_O_6_), (C_16_H_14_O_5_), (C_17_H_14_O_5_), and (C_16_H_10_O_6_) was also shown by high-performance liquid chromatography and ultra-high-performance liquid chromatography-quadrupole time-of-flight mass spectrometry studies. Because these degradation products were created by the elimination of AFB1's partial hazardous sites, they may be less harmful than AFB1 itself, based on the structure-activity connection. Detoxification of AFB1 in food stuff, *P. aeruginosa* M-4 has good potential [170].

#### 4.4.5 Organic acids

Three food-grade organic acids are used to detoxify the AFs in nuts. For the detoxification of aflatoxin B1 (AFB1) and total aflatoxins (TAFs), which include AFB2, AFB1, and AFG2, in a variety of nuts, including almond, peanut, pistachio, and walnut, at two different moisture levels (10.3 and 16 3 %), citric, lactic, and propionic acid aqueous solutions are used at five different concentrations (1, 3, 5, 7 and 9 %). For the purpose of determining the qualitative and quantitative levels of AFs, high-performance liquid chromatography (HPLC) in conjunction with a fluorescence detection technique was used. The findings demonstrated that raising the acid concentration considerably improved AFB1 and TAF decontamination in contaminated nuts. Furthermore, AFB1 was transformed into less harmful compounds known as AFD1 by hydrolysis of the lactone ring after being exposed to citric and lactic acids. Furthermore, of the three organic acids, citric acid was shown to be the most effective in destroying TAFs. The outcomes of the current investigation were superior to those of standard approaches for AFs detoxification [171].

## 5 Ochratoxin

The most prevalent mycotoxins, ochratoxin, is a serious issue for the food stuff. Ochratoxin A (OTA), a member of the ochratoxins family, is dangerous, persistent, and naturally present in food. The inclusion of a dihydro isocoumarin chlorine atom in the structure of OTA sets it apart from ochratoxin B. The name for the OTA ethyl ester derivative is ochratoxin C. Additionally, ochratoxin alpha is the name for the non-amide component of OTA and OTC, whereas ochratoxin is the name for the component of OTB.

Multiple harmful effects of OTA are identified in animal cell lines.OTA targets the kidney and causes nephropathy. OTA was classified by the International Agency for Research on Cancer (IARC) as a category 2B human carcinogen in 1993. Additionally, exposure to OTA frequently leads to genotoxic, hepatotoxic and carcinogenic effects [172], [173].

### 5.1 Source

Fungi belonging to the genera *Aspergillus* and *Penicillium* produces Ochratoxin A (OTA) which is commonly found in food and agricultural products, causing significant loss in the economy as well as to human health [174].

### 5.2 Occurrence

Milk and over 90 other different plant- and animal-derived foods to OTA dietary exposure. Cereals, olives, beans, alcoholic beverages, coffee, chocolate-based goods, raisins, figs, licorice, pulses, pumpkin seeds, and tea are all sources of OTA. Ochratoxins (OTs) contaminate different types food by infecting crops before to and after harvest. The most dangerous OT produced is ochratoxin A (OTA), associated with neurotoxicity and carcinogenesis [173].

### 5.3 Toxicokinetics

OTA, a prevalent mycotoxin that is frequently detected as a maize. It has been demonstrated to be immunotoxic, teratogenic, nephrotoxic, and hepatotoxic. OTA produces kidney and liver tumours has been demonstrated by Tao et al. [174].

Its action's biochemical and molecular components were initially investigated in bacteria. The emergence of "magic spots" (ppGpp and pppGpp) indicated a reduction in the amino acid loading of transfer ribonucleic acids (tRNA). The recent discoveries showed that ochratoxin A inhibits bacterial, yeast, and liver phenylalanyl-tRNA synthetases supported this theory. Phenylalanine competes with the inhibition, and an excess of this amino acid reverses it. As a result, protein synthesis is decreased, as demonstrated by hepatoma cells in culture, the far more sensitive Madin Darby canine kidney cells, and in vivo mouse liver, kidney, and spleen tissues—the inhibition being more potent in the latter two tissues. In cell cultures and in living organisms, an excess of phenylalanine also inhibits the suppression of protein synthesis. The aminoacyl tRNA synthetases that are unique to each amino acid show identical inhibitory effects when phenylalanine is substituted in ochratoxin A analogues [175]. Nourbakhsh and Tajbakhsh [176] has reported the possible mechanism involved for the neurotoxicity, might involve oxidative DNA, protein and lipid damage, and apoptosis. However, the actual mode of action of OTA seems to be very complex and not yet understood clearly [177].

### 5.4 Detoxification strategies

#### 5.4.1 Traditional methods

The conventional method for the removal of OTA like the use of control and preventive measures at various stages of the production like harvesting, storage and transport are effective up to certain extent in controlling and managing the production of OTA. Traditional method also includes the application of physical and chemical treatment during the food production process. The physical method includes the application of the radiation, washing, thermal, peeling and the use of OTA adsorbing materials. The chemical approach includes the application of the synthetic fungicides which generates the toxic residues in the food. The use of substances like ammonium bisulfides, ozone, and alkaline compounds which hydrolyses the OTA [172], [173].

#### 5.4.2 OTA detoxification by plant extracts/active compounds

The utilization of plant extracts as environmentally safe ways to detoxify OTA is growing because of their potent antifungal and detoxifying bioactive components [178]. Polyphenols, flavonoids, and essential oils are among the extracts that interact with OTA to lessen its toxicity [179]. Known for its bioactive components, eucalyptus extract has the potential to be used in crop management because it can suppress OTA formation in grapes by up to 85.75 % [180]. Antioxidants and organosulfur compounds found in common household spices like ginger and garlic break down OTA and support detoxification pathways [181]. Research has demonstrated notable decreases in the levels of OTA in food items, suggesting that they could be incorporated into procedures for handling and storing food. Since extracts of ginger and garlic have been demonstrated to have lower levels of toxins at harvest, employing plant extracts as preharvest treatments in agriculture can reduce OTA contamination in crops [182].

#### 5.4.3 OTA detoxification using nanoparticles

Activated carbon and graphene oxide are examples of high-surface area nanoparticles that may efficiently absorb OTA from contaminated materials, lowering its bioavailability [163], [183]. Silver and zinc oxide nanoparticles have antifungal qualities that stop the growth of mold and stop the creation of OTA [156], [184]. Magnetic nanoparticles make it simple to separate from food items, lowering the possibility of contamination. These techniques have been used in a number of industries, such as animal feed and food processing [185], [186]. Nanomaterials in food packaging improve safety and prolong shelf life by preventing the formation of mold and OTA [187]. By lowering OTA absorption in ruminants and poultry, plant extracts or nanoparticles in animal feed can stop poisons from getting into the human food chain. All things considered, nanotechnology presents encouraging options for OTA detoxification across a range of sectors [188].

## 6 *T-2 toxin*

T-2 toxin is one of the most common and toxic trichothecene mycotoxins. T-2 toxin is the most toxic trichothecene mycotoxin, and it exerts potent toxic effects, including immunotoxicity, neurotoxicity, and reproductive toxicity. Recently, many novel compounds including 3′,4′-dihydroxy-T-2 toxin and 4′,4′-dihydroxy-T-2 toxins have been discovered [189].

### 6.1 Source

T-2 toxin belongs to the type A trichothecene, produced by *Fusarium* species (F. *poae, F. sporotrichioides*, and *F. tricinctum*) [142]. In vitro the strains of *Fusarium* produce high level of T-2 toxin when supplemented with vermiculite. Fusarium inoculated over the modified Gregory medium supplemented with 2 % soya meal, 10 % glucose and 0.5 % corn step liquor, produced maximum T-2 toxin when incubated at 19 ℃ for 24 days [190].

### 6.2 Occurrence

Cereals like maize, rice, barley, wheat, oats and soy beans and their derivatives are mostly affected by T-2 toxin, among this maize is the most vulnerable crop for T-2 contamination in Croatia [191]. The T-2 toxin has a very limited half-life and are flushed out of the body within 48 hours, but this time is greatly influenced by the amount and rout of exposure. On ingestion to humans, the thiol group of T-2 toxin inhibits the translation process by binding itself to the peptidyl transferase enzyme and targeting the 60 s ribosomal unit [192].

### 6.3 Toxicokinetics

In the past 10 years, it has collected impressive consideration because of its powerful neurotoxicity as it can cross the blood brain barrier (BBB) and can accumulate itself in the CNS. In-vitro studies along with the animal model studies represents that the T-2 toxin induces ROS and oxidative stress to cause neurotoxicity as well as mitochondrial dysfunction by affecting crucial pathways like p53, Akt/mTOR, MAPK, NF-κB and PKA/CREB. In addition to this, T-2 toxin also affect the mitochondrial respiratory chain complex as well as mitochondrial biogenesis. Certain antioxidant like N-acetylcysteine (NAC) contributes towards the protective effects via activation of Nrf2/HO-1 and autophagy [193].

T-2 toxin is metabolized with enzymes like CYP3A4 and carboxylesterase to produce 3’-hydroxy-T-2 toxin and HT-2 toxin as primary product. T-2 toxin H is its modified form elicit the immunotoxin activity via JAK/STAT pathway. The intestinal microbiota enhances the toxicity by hydrolysing to form T-2–3glucoside causing the autophagy, hypoxia-inducible factors and exomes mediated T-2 toxin induced immunotoxicity. T-2 induced autophagy promotes immunosuppression initiated within the cell resulting the pathogen to evade the host immune response [189].

The harmful impact of T-2 on poultry incorporates cytotoxicity, genotoxicity, metabolism modulation, immunotoxicity, etc., T-2 toxin primarily led to cytotoxicity characterized by the protein and nucleic acid synthesis inhibition, cell cycle alteration leading to the oxidative stress apoptosis and necrosis, damaging the vital organs like liver, digestive tract, bone, kidney etc., resulting in the loss of function of these organs. Some of the preventive mechanism includes Glutathione redox system, superoxide dismutase, catalase and autophagy [194].

### 6.4 Detoxification strategies

#### 6.4.1 Microbial detoxification

Bacterial communities isolated from 17 of 20 samples of soils and waters with generally different geological starting points used T-2 toxin as a sole wellspring of carbon and energy. *Rhodotorula rubra* bioassay suggest that the isolates detoxify T-2 toxin by the cleavage of the side chain of acetyl moieties and produces HT-2 and T-2 triol toxin. Apart from this, conversion of neosolaniol and thence to a low toxic compound 4-deacetyl neosolaniol, which also gets further degraded by some bacteria communities like TS4 and KS10 [194].

#### 6.4.2 T-2 detoxification using plant extracts/Phytochemicals

Plant extracts are being recognized for their bioactive components, which have the ability to detoxify T-2 toxin. These chemicals, which include flavonoids and phenolics, can interact with the T-2 toxin, rendering it inert and limiting absorption in the gastrointestinal system. They also include antioxidants, such as curcumin in turmeric and polyphenols in green tea, which can help reduce oxidative stress produced by T-2 toxin exposure [195], [196]. Antioxidant selenium reduced the liver damage caused by T-2 toxin and prevented ferroptosis. Iron death and oxidative damage were decreased by Na_2_SeO_3_ treatment, opening the door for its everyday application [197].

Polyphenol menthol was used to detoxify T-2 toxin induced inflammatory action in vitro method demonstrated by Rachitha et al. [198]. Further in vivo studies on Menthol also confirms the amelioration of T-2 toxin induced dermal inflammation in mice model [199].

#### 6.4.3 T-2 detoxification using nanoparticles

Antifungal nanoparticles, such as silver and zinc oxide, prevent the development of Fusarium species that produce T-2 toxin, reducing the risk of toxin formation during agricultural storage and processing [200]. Magnetic separation uses a magnetic field to detoxify and easily remove from treated substrates, resulting in a considerable reduction in T-2 toxin [201].

Food packaging contains nanoparticles that actively absorb mycotoxins, extending shelf life and improving food safety. Animal feeds containing detoxifying plant extracts or adsorbent nanoparticles can protect animals from mycotoxin exposure, hence boosting health and production. Nanotechnology may also be used in polluted soil or water to efficiently absorb and breakdown T-2 toxin, providing a viable alternative for recovering agricultural land contaminated with mycotoxins [200].

#### 6.4.4 Other agents

As the most toxic trichothecenes, T-2 toxin causes severe damage to multiple organs, especially to liver by the various mechanisms like oxidative stress, DNA methylation, mitochondrial damage, autophagy and apoptosis. Once the infected food item gets absorbed by the intestine the toxicity of the toxin gets reduce to a certain level into the blood as the prime target is the liver [142].

Poultry feeds containing T-2 toxin can be detoxified using various agents such as adsorbing agents (Ex., Aluminosilicate-based clays and microbial cell walls), bio-transforming agents (Ex., *Eubacterium* sp. BBSH 797 strain), and indirect detoxifying agents (Ex., Plant derived antioxidant), however, multi-component detoxifying agents can provide much more protection against T-2 toxin [118].

## 7 Deoxynivalenol (DON)

DON is a foodborne mycotoxin which is naturally present in the grains, cereals and cereal based foods during the preharvest, processing, drying and storage. Detoxification of DON is quite difficult as it is extremely thermostable and can withstand high temperature ranging from 170 to 350℃ and causes unusual reduction in body weight, immunotoxicity and reproductive defects [202], [203].

### 7.1 Source

DON, is produced by *Fusarium* species, one of the most common occurring microorganisms. DON causes acute as well as chronic toxicity when ingested, and the symptoms include abdominal pain, diarrhea, salivation, vomiting, anorexia, malaise weight loss and change in dietary efficacy [143].

### 7.2 Occurrence

The primary occurrence of these mycotoxins is reported in almost all cereal grains and fresh agro-products.Wheat, oats, maize and barley are mostly get contaminated with DON. The toxin causes significant economic loss by impaired growth among farm animals while harmful effects such as nephrotoxic, hepatotoxic, and genotypic effects in humans have been reported because of complex structural formation during degradation/acetylation reaction [204], [205].

### 7.3 Toxicokinetics

DON, being the most widespread mycotoxin, it posses great health threat for the human as well as farm animals, with pigs to be the most susceptible followed by the mice/rats and poultry to be the least susceptible. DON gets absorbed fast and can be widely distributed among multiple organs with the first enrichment takes place in the plasma, liver and subsequently gets accumulated in to the intestine. The effect of DON varies greatly among different animals which can be attributed with the presence of detoxifying gut microbiota and the clearance time taken by the toxin. Pigs and human being the most susceptible have the absorption rates of about (1 hr < tmax < 4 hr), high bioavailability (>55 %) and long clearance time (2 hr < t1/2 < 4 hr). Mice and rats have the similar absorption (tmax< 0.5 hr) and clearance (t1/2 <1 hr), however, in rats’ major proportion of DON gets excreted in the form of DOM-1, this suggests the importance of the gut microbiota. Poultry are the least sensitive to DON because of their fast absorption rate (tmax <1 hr), low oral bioavailability (5–30 %), and shorter clearance time (t1/2 <1 hr). In comparison to the land animals, aquatic animals show significantly slower plasma clearance [206].

### 7.4 Detoxification strategies

#### 7.4.1 Biological methods

One of the methods for the detoxification of DON is by using the biological sources such as microbes or enzymes as some of the microorganisms have the inherent ability to absorb and degrade it before it could get absorbed by gastrointestinal organ [205].

Detoxification of DON by physical and chemical method may lead to deteriorate the nutritional value, safety as well as the palatability, whereas, detoxification based on microbial strains or enzymes has added advantages as they are highly specific, have high efficiency and avoid generation of any kind of secondary pollution [207].

The virulency of the DON can be minimized by the production of transgenic variety of the food. The scientists have discovered a principal mechanism in barley for the tolerance of DON toxin is its glycosylation by specific uridine diphosphate-dependent glucosyltransferases (UGTs) producing DON-3-β-D-glucoside (D_3_G). In order to test this, transgenic durum wheat and bread wheat plant expressing the barley gene HvUGT13248 in the flower tissue. This has showed a considerable DON to D_3_G conversion ability and decrease in DON+D_3_G content in the flour. The transgenic bread wheat also exhibited a UGT in a dose dependent efficacy for the DON detoxification. In addition to this, the transgenic wheat showed resistance to both Fusarium Head Blight (FHB) and Fusarium Crown Rot (FCR) both caused by the f*usarium* species and produces DON toxin as virulence factor during pathogenesis. The transgenic wheat expressing HvUGT13248 gene resulted in the reduction of FHB and FCR to an extent of 50 %, suggesting that the DON detoxification can be considered as an important trait for the wheat breeding programme which can target FHB and FCR resistance [208].

#### 7.4.2 DON detoxification using plant sources/phytochemicals

Plant extracts varied phytochemical profiles have led to an increase in their usage in mycotoxin detoxification. The harmful effects of DON can be lessened by these extracts capacity to bind to it and produce non-toxic complexes that decrease its bioavailability. They also have antioxidant qualities, including the polyphenols in green tea and turmeric's curcumin, which strengthen detoxification pathways and shield cells against DON-induced damage [209]. Glutathione S-transferase is one of the detoxification enzymes that some extracts can cause to conjugate DON and aid in its removal. Certain plant extracts have the ability to increase DON's vulnerability to metabolic processes, changing it into less dangerous forms [210]. Abdel-Wahahnab and El-Nekeety [211] investigated silymarin and inulin as antioxidants for preventing DON-induced oxidative damage. Plant extracts, such as those from garlic and ginger, can lower DON levels in crops, reducing fusarium infections and the resulting generation of toxins. This has been demonstrated in agricultural operations [212], [213].

#### 7.4.3 DON detoxification using nanoparticles

Several nanoparticles have antifungal characteristics that prevent Fusarium species from developing and minimize fungus-produced toxins [214].

Food processing contamination hazards are reduced and the detoxification process is made simpler by magnetic nanoparticles [215]. Nanotechnology may also be used in food packaging systems to stop the growth of fungi and lower the generation of mycotoxin. This improves storage safety and prolongs shelf life. By lowering the bioavailability of DON, detoxifying plant extracts or nanomaterials can be used into animal feed formulations to improve livestock health and keep mycotoxins out of the human food chain [163]. In order to handle residual contamination, remove DON from polluted soil and water, and shield agricultural areas from further infection, nanotechnology can also be used in environmental decontamination procedures [216].

## 8 Fumonisins

Fumonisins are the group of naturally occurring toxins discovered in 1988, among which Fumonisin B1 (FB1) is considered as the most potent mycotoxins produced mainly by *Fusarium proliferatum* and *Fusarium verticillioide.* FB1 causes extensive contamination mainly in corn, rice, wheat and their products world-wide posing a great health risk and toxicity to both animal and humans. FB1 causes oxidative stress, endoplasmic reticulul stress, cellular autophagy and apoptosis [144], [217].

### 8.1 Toxicokinetics

The toxicity of fumonisins are largely species specific, and upon exposure causes neurological disorders among equids, pulmonary edema mong swine, esophageal cancer in humans and both kideney and liver toxicity in rodents [218]. It also causes variety of toxicity including autophay, apoptosis, neurotoxicity, reproductive toxicity, tissue and organ toxicity and carcinogenicity in other organisms. Many reports also suggest that the toxicity caused by the fumonisins is by the modulation of sphingolipid metabolism and induction of oxidative stress [219]. It has also been found that the FB1 has the potential to cause systemic toxicity, including neurotoxicity, hepatotoxicity, nephrotoxicity and mammalian cytotoxicity [220].

### 8.2 Detoxification strategies

Enzymatic detoxification has been proven to the best method for the detoxification, but it is limited by the serious shortage of the detoxification enzymes. Based on the data mining and Liquid Chromatography Mass Spectrometry (LCMS), a novel enzyme carboxylesterase FumDSB from *Sphingomonadales* bacterium expressing in *E. coli* can catalyse the production of hydrolyzed fumonisin B1 from FB1. The enzyme FumDSB has the high sequence novelty, expressing only 34 % of similar sequence identity with the other three carboxylesterases found to be important for fumonisin detoxification. Beside this, FumDSB has also shown high degradation activity at an optimum temperature of 30 – 40 ℃, over a broad pH range varying from 6 to 9, making it ideal for the use in the animal physiological condition [221].

Another enzyme, transaminases also showed the promising result for the significant reduction in the FB1 toxicity. Data mining and characterization of enzyme has provided three novel fumonisin detoxifying transaminases FumTSTA, FumUPTA and FumPHTA, sharing only 61 – 74 % similar sequence identity. Moreover, the recombinant protein showed good pH stability with diverse pH reaction range and thermostability [222].

*Pseudomonas* genus (bacterial consortium SAAS79) has been identified with the help of antibiotic-based selection and 16 rDNA sequencing, has the potential to degrade 90 % of FB1 at concentration of 10 µg/mL within 3hrs at an optimal temperature of 28–35 ^o^C by the enzyme produced from the intracellular spaces [223].

#### 8.2.1 Fumonisin detoxification using plant sources/phytochemicals

According to studies, several phenolic compounds found in plant extracts have the ability to break down fumonisins or prevent their formation. Because of their antioxidant qualities, these substances counteract the reactive oxygen species that fumonisins produce. According to experiments, several phenolic compounds can decrease the formation of fumonisin B1 and so increase grain safety [179]. Extracts from *Lippia nodiflora* have shown notable detoxifying levels in a matter of minutes. Potential uses for plant extracts in agriculture include animal feed and grain storage systems [224].

#### 8.2.2 Fumonisin detoxification using nanoparticles

By employing nanoparticles with certain qualities, nanotechnology offers a possible means of detoxifying mycotoxins, including fumonisins. Because of their large surface area and reactivity, magnetic nanoparticles make it easier for fumonisins to physically adsorb from contaminated food matrices [225]. Effective elimination of toxins can result from functionalization with certain ligands, which can increase their binding efficacy. Silver nanoparticles have been shown to be efficient in preventing fungal development and lowering the levels of mycotoxin in cereal goods, according to a wealth of research on nanoparticles in food processing [226]. Due to their ease of separation from food matrices after treatment, magnetic nanoparticles are a viable option for industrial applications. The high cost and scalability of nanotechnology solutions may restrict their use in developing areas where fumonisin contamination is prevalent. However, these issues are being addressed by improvements in manufacturing methods. Regulatory acceptance is crucial for integrating nanotechnology into food safety regulations [164], as testing and regulatory review are time-consuming [227], [228]. Public perception may be reluctance to accept nanotechnology due to safety and environmental concerns, so transparency and public education about the benefits and safety of these technologies are essential for acceptance [229].

## 9 Comparative analysis of traditional vs. Novel detoxification methods

Mycotoxins such as aflatoxin, ochratoxin, T-2 toxin, fumonisin, and deoxynivalenol have been treated using traditional detoxification procedures such as physical separation, heat treatment, and chemical decontamination. These approaches can lessen contamination but may not address existing poisons in the crop. Heat treatments are restricted since many mycotoxins are heat resistant. Chemical techniques such as ammonia treatment or organic acids can destroy mycotoxins, however they may leave residues or have an impact on the nutritional quality of the finished product. Traditional approaches are frequently hampered by regulatory constraints and customer concerns about chemical residues [230].

Novel detoxification technologies, such as biological detoxification and nanotechnology-based systems, provide increased safety and selectivity in lowering toxins in food or feed. Biological approaches employ bacteria such as *Lactobacillus* or *Aspergillus* to breakdown toxins into less harmful or non-toxic metabolites, whereas nanotechnology break throughs show promise for precisely binding and eliminating mycotoxins. These methods are ecologically friendly and improve food safety and nutritional value. However, practical implementation confronts problems such as scalability, expense, and regulatory approval, making it a supplement to existing approaches in the medium term [146].

## 10 Statistical overview of mycotoxin contamination

The mycotoxin contamination present in the food is a matter of global concern. A meta- analysis report in 2023 has reported the presence of detectable level of atleast one mycotoxin in over 60 % of the tested food sample, aflatoxin being the most prevalent. The report also confirms with the presence of aflatoxin in approximately 30 % of the maize samples tested in sub-Saharan Africa, with exceeding the permissible limits set European Union which results in significant economic loss and adverse health impact [231], [232], [233], [234].

In Asia, rice contamination by fumonisins and deoxynivalenol has raised similar safety concerns. In India, out of total rice sample tested, approximately 40 % showed higher level of fumonisins, increasing the health risks like gastrointestinal issues. Similarly, heavy metal contamination of maize and cassava by aflatoxins in Southeast Asia contributing towards the concern for local food stuffs. In Latin America, fumonisins have been identified in more than half of maize-based items in certain districts, adding to an ascent in esophageal malignant growth cases. Also, irregular episodes of ochratoxins and T-2 poisons have happened in inappropriately put away cereals, featuring the requirement for watchfulness away and handling practices [235], [236], [237], [238].

In Latin America, fumonisins have been detected in over 50 % of maize-based products in some regions, contributing to a rise in esophageal cancer cases. Similarly, sporadic outbreaks of ochratoxins and T-2 toxins have occurred in improperly stored cereals, highlighting the need for vigilance in storage and processing practices [239].

Contrastingly, Europe and North America report lower contamination levels due to stringent regulatory frameworks, though occasional outbreaks persist. These findings emphasize the necessity of enhanced surveillance systems, region-specific mitigation strategies, and heightened awareness among stakeholders to address the global challenge posed by mycotoxins effectively [240].

Global surveys reveal alarming levels of mycotoxin contamination in food supplies. A comprehensive 2023 meta-analysis reported that over 60 % of tested food samples contained detectable levels of at least one mycotoxin. Among these, aflatoxins were found to be the most prevalent, particularly in warm and humid regions. For example, in sub-Saharan Africa, approximately 30 % of maize samples exceeded the permissible aflatoxin limits set by the European Union, causing significant health and economic repercussions [234].

In Asia, rice contamination by fumonisins and deoxynivalenol has raised similar safety concerns. For instance, a study conducted in India indicated that over 40 % of rice samples exceeded fumonisin thresholds, increasing health risks such as gastrointestinal illnesses. Similarly, in Southeast Asia, heavy contamination of maize and cassava by aflatoxins has highlighted the vulnerability of local food systems [241].

Latin America faces parallel challenges, with fumonisins detected in over 50 % of maize-based products in some regions, contributing to a rise in esophageal cancer cases. In contrast, Europe and North America have reported lower contamination levels, thanks to stringent regulatory frameworks, but sporadic outbreaks continue to occur, particularly with ochratoxins in grains and T-2 toxins in improperly stored cereals [242].

## 11 Case studies

1. Kenya, 2004: A severe aflatoxicosis outbreak linked to contaminated maize resulted in over 300 illnesses and 125 deaths. This case highlights the urgent need for effective storage and monitoring systems, especially in rural areas where infrastructure is lacking [243].
2. India, 2020: High levels of aflatoxin M1 in milk were detected, raising concerns about the indirect exposure of consumers through animal-derived products. This incident emphasized the importance of monitoring feed quality in dairy farms and implementing strict quality control measures [244].
3. Serbia, 2012: Ochratoxin A contamination in cereals was linked to prolonged drought conditions, underscoring the role of climate change in mycotoxin prevalence. This event triggered discussions on incorporating climate resilience into agricultural practices [245].
4. Nigeria, 2018: Contaminated groundnuts exported to the European Union were rejected due to aflatoxin levels exceeding permissible limits. This led to economic losses for farmers and highlighted the need for better pre-harvest and post-harvest practices [246].
5. United States, 2013: A fumonisin outbreak in maize-based products led to increased cases of esophageal cancer in affected regions. Enhanced surveillance programs and early-warning systems were recommended to mitigate future occurrences [51].
6. Philippines, 2016: An outbreak of deoxynivalenol in rice caused widespread gastrointestinal distress among consumers, particularly affecting school-aged children. The incident underscored the importance of investing in toxin-resistant crop varieties and public education on food safety [247].
7. Russia, 2010: Severe contamination of grains with T-2 toxin was reported following prolonged wet and humid conditions during harvest. This led to widespread livestock mortality and instances of alimentary toxic aleukia (ATA) among humans, characterized by hemorrhaging and immune suppression.
8. Ukraine, 2014: During a period of political and economic instability, poorly stored grains showed high levels of T-2 toxin contamination. This resulted in acute poisoning cases in rural areas where access to food testing and healthcare was limited, emphasizing the need for robust food security frameworks [190].
9. China, 2018: A study documented high levels of T-2 toxin in cornmeal products, which led to gastrointestinal and hematological disorders among affected populations. The incident spurred research into cost-effective detection technologies and improved awareness campaigns [110].

## 12 Regulatory framework

Guidelines connecting with mycotoxins have been laid out in numerous nations to protect the customer from the impacts of mycotoxins. Various factors paly an important role in the limiting the use of mycotoxins. The factors, for instance the accessibility of toxicological information and event information, itemized information about opportunities for examining and examination, and financial issues. Toward the end of 2003, roughly 100 nations (covering around 85 % of the world's occupants) had explicit guidelines or definite rules for mycotoxins in food. The guidelines were connected with aflatoxins (B1, B2, G1 and G2), aflatoxin M1, trichothecenes (deoxynivalenol, diacetoxyscirpenol, T-2 poison and HT-2 poison), fumonisins (B1, B2, and B3), agaric corrosive, ergot alkaloids, ochratoxin A, patulin, phomopsins, sterigmatocystin, and zearalenone. In Europe, and specifically in the Europian Union (EU), administrative and logical interest in mycotoxins has gone through an improvement somewhat recently from independent public movement towards more EU-driven action with a primary and organization character. Orchestrated EU restricts now exist for 40 mycotoxin-food mixes. It is normal this number will fill in 2007 to roughly 50. The immediate or circuitous impact of European associations and projects on the EU mycotoxin administrative advancements is huge. They incorporate the European Sanitation Authority, the Logical Participation on Questions connecting with Food, the Quick Ready Framework for Food and Feed, the making of an EU People group Reference Research facility for Mycotoxins and a command of the EC to the European Normalization Board of trustees in strategies for examination for mycotoxins in food. Enormous container European exploration and systems administration projects as "BioCop" and "MoniQA" are likewise significant [248].

## 13 Conclusion and future perspectives

Mycotoxins in food and feed represent a huge danger to quality, security, and purchaser wellbeing. A basic survey recommends a shift from focused on to untargeted screening investigations to all the more likely comprehend openness levels and possible poisonousness. As the fungus are omnipresent in the environment and the food and the feeds are more susceptible for the fungal infection and mycotoxin contamination. It is very important to manage the mycotoxin present in the food as the contamination can occur at any stage of the production as well as most of the mycotoxins are heat stable and can withstand the harsh environment during the processing phase also and has the potential to cause a great loss to the health of human as well as the farm animal along with a great impact on the economy by affecting the entire agri-food supply chain. This review outlines the primary mycotoxins and their effects on human and animal health. The use of the biological source such as different strains of bacterium, enzymes and identification of the novel resistance gene and incorporation of these genes for the production of the transgenic food can be considered as a safe, environmentally friendly with least alteration effect over the nutritive values of the food stuff. LC-HRMS is reasonable for recognizing numerous mycotoxins and changed mycotoxins in food and feed items, however legitimate measurement and location of concealed/stowed away mycotoxins in complex lattices stay a significant snag. omics innovations are arising as an important instrument to exhaustively investigate the impact of mycotoxins on organic entities by examining different biofluids. Untargeted metabolomics permits analysts to actually look at significant changes in biochemical pathways and the method of activity of various mycotoxins. HRMS joined with an omics approach could assist administrative bodies with restricting mycotoxins in food and feed. Notwithstanding, the absence of exact standard working methodology makes results challenging to look at.

## CRediT authorship contribution statement

**Raghavendra Vinay B:** Validation, Formal analysis. **P Rachitha:** Writing – review & editing, Writing – original draft, Validation, Supervision, Methodology, Conceptualization. **Shekhar Ravikant:** Writing – original draft, Software.

## Declaration of Competing Interest

The authors declare that they have no known competing financial interests or personal relationships that could have appeared to influence the work reported in this paper.

## Data Availability

Data will be made available on request.
